# Nutritional Composition of Biscuits from Wheat-Sweet Potato-Soybean Composite Flour

**DOI:** 10.1155/2022/7274193

**Published:** 2022-06-09

**Authors:** Ponka Roger, Bisso Monesso Marvist Bertrand, Zomegni Gaston, Bissada Nouhman, Fokou Elie

**Affiliations:** ^1^Department of Agriculture, Livestock and Derivated Products, National Advanced School of Engineering of Maroua, University of Maroua, Cameroon; ^2^Department of Textile and Leather Engineering, National Advanced School of Engineering of Maroua, University of Maroua, Cameroon; ^3^Institute of Agricultural Research for Development, Maroua, Cameroon; ^4^Department of Biochemistry, Faculty of Science, University of Yaoundé I, Yaoundé, Cameroon

## Abstract

The aim of this study was to evaluate the nutritional composition of biscuits from wheat-sweet potato-soybean composite flours. Substitutions of wheat flour with sweet potato and soybean flours at the rate of 25% (wheat flour 375 g, sweet potato flour 100 g, and soybean flour 25 g) (T1), 50% (wheat flour 250 g, sweet potato flour 200 g, and soybean flour 50 g) (T2), 75% (wheat flour 125 g, sweet potato flour 300 g, and soybean flour 75 g) (T3), and 100% (wheat flour 0 g, sweet potato flour 400 g, and soybean flour 100 g) (T4) were made to obtain the wheat-sweet potato-soybean composite flours. Wheat flour without sweet potato and soybean flour was used as control (T_0_). The functional properties (water absorption capacity and water solubility index) of the flours were determined. Sensory evaluation of biscuits was determined. The proximate and mineral contents of the most preferred biscuits were determined. Results showed that the substitution significantly increases the functional properties of the flours (*P* < 0.05). Sample 75% (T3) is the most preferred biscuit. The incorporation of sweet potato and soy flour in the formulation significantly increases the moisture, fat, protein, fiber, copper, manganese, calcium, magnesium, and potassium contents of the biscuits (*P* < 0.05). Biscuit 75% (T3) records the highest levels in protein and fiber while sample T_0_ (control) records the highest levels of iron, zinc, and sodium. In terms of overall acceptability and nutrient contents, sample 75% (T3) is the best biscuits.

## 1. Introduction

The consumption of wheat products especially bread and biscuits occupies a prominent place in the diet of populations, even in non-wheat-producing countries. The latter are becoming more and more dependent on nations of wheat producers, particularly during economic crises where wheat is very expensive to import. This cereal is imported while our local products remain unexploited and are decaying [1].

World wheat production is around 580 million tons per year, of which 350 million tons are processed into flour and meal. In Cameroon, since the year 2000, the quantities of wheat imported have increased sharply. Until 2007, they were around 280,000 tons. From 2008, there was a significant increase in these quantities of which 681,778 tons were imported in 2017 at a cost of around 103.7 billion francs CFA with an average price of around US $300 per ton. The unit import price increased to 3.3%.

In Cameroon, the attempt to introduce wheat cultivation has not produced the expected results. However, to reduce the use of imports of wheat flour and to improve the value of local products, the solution is to integrate the tubers (sweet potatoes and cassava) in manufacturing of bread wheat flour when producing biscuit. The tubers have not yet undergone a significant modern transformation, due to their low value. To remedy this situation, technologies are increasingly being developed to replace wheat flour with flour obtained from local food resources [2]. Sweet potato is one of the major crops in the world. On the one hand, because of its short cycle, its abundance, and its cheap cost, unlike other tubers, it constitutes a potential food for populations with limited resources. It is cultivated in 114 countries of the world with a production totally in 2006 to approximately 123 million tons on a total cultivated surface of approximately 9 million hectares [3]. It is an important plant in tropical and subtropical zones, the increase in its production and consumption constitutes a nutritional advantage for the inhabitants of these regions.

Also, sweet potato is an important source of carbohydrates (96%), in the form of simple carbohydrates and dietary fibers, which plays an important role in energy deficiencies [4]. Apart from carbohydrates, sweet potato is a good source of vitamins A and minerals essential for the proper functioning of the organism. Its high vitamin A content up to 4000 IU per 100 g of tubers depending on the variety is much more important than that of other roots and tubers, and its vitamin C content (30 mg/100 g) is also remarkable [5]. In fact, in developing countries, research efforts have partially been made to replace wheat flour with flour from available local products [6]. This is in order to reduce costly imports of wheat and increase the use of local products [7]. Attempts to replace wheat flour with flour made from local rich products in carbohydrates such as cereals (corn, rice, sorghum, and millet), tubers (cassava, taro, sweet potato, and yam), and rich in proteins (cowpea, soybeans) have been conclusive [8].

In Cameroon, the annual production of sweet potato is 288,970 tons and that of soya is 12,544 tons [9]. These local products can also be used in the food industry to make cakes, bread, biscuits, etc. Thus, the aim of this study was to evaluate the nutritional composition of biscuits from wheat-sweet potato-soybean composite flours.

## 2. Materials and Methods

### 2.1. Raw Materials

Sweet potato (*Ipomoea batatas*), soybean (*Glycine max*), and other ingredients were collected from the local market, Maroua, a town in the Far North region of Cameroon.

### 2.2. Preparation of Raw Materials

#### 2.2.1. Preparation of Sweet Potato Flour

The potato tubers were manually peeled, washed, and immediately soaked in water to prevent browning. After draining and cutting, they were cut into thin strips and dried in an oven at 60°C for 12 hours. The dried tubers were milled into flour. The flours were screened through a 0.25 mm sieve.

#### 2.2.2. Preparation of Soy Bean Flour

Soybean seeds were sorted and soaked in water for 12 h. Thereafter, the seed coat was removed and drained. The seeds were then cleaned and boiled for 30 min and dried at 65°C for 9 h. The dried seeds were milled into flour. The flours were screened through a 0.25 mm sieve.

### 2.3. Functional Property Determination of Flour

#### 2.3.1. Water Absorption Capacity

The water absorption capacity of the studied flours was determined according to the method of Beuchat [10]. A sample of 1 g was mixed with 10 mL of distilled water, and the mixture was stirred for 30 min and centrifuged at 5000 rpm for 30 min in a HIMAC brand centrifuge. The pellet was recovered, weighed (*M*1), and dried in an oven at 105°C for 24 h. The dried pellet weight (*M*2) was determined. The water absorption capacity (WAC) was calculated as follows: WAC = (*M*1 − *M*2) × 100/*M*2. All samples were analyzed in triplicate.

#### 2.3.2. Water Solubility Index

The water solubility index (WSI) was determined according to Anderson et al. [11]. WSI reflects the importance of starch degradation. It is calculated as WSI = (*M*0 − *M*1)/*M*0 × 100, where *M*0 is the mass of sample and *M*1 is the final mass after dissolution in water. All samples were analyzed in triplicate.

### 2.4. Preparation of Biscuits

#### 2.4.1. Quantities of Each Ingredient Used in the Preparation

The quantities of each ingredient used in the preparation are presented in Table 1.

#### 2.4.2. Elaboration of the Biscuit Cream

After weighing the ingredients, the margarine and sugar were mixed using a manual thresher to give a cream. The beaten egg was incorporated into the cream, and then, the mixing continues until a snow-white paste was obtained.

#### 2.4.3. Preparation of the Biscuit Dough

The ingredients and raw materials in powder form previously prepared (salt, flour, and yeast) were added to the cream and the whole were manually mixed. Water was added until the dough was smooth. After kneading, the dough was spread on the pastry board using the rolling pin. The display thickness was fixed at 4 mm for better cooking. This thickness was measured using a fabricated frame.

#### 2.4.4. Baking Biscuit Dough

The oven was preheated to 180°C. The biscuit dough pieces were placed on the baking trays and placed in the oven at a temperature between 140 and 150°C. The temperature was set manually. This operation allowed us to obtain good quality biscuits by adjusting the baking temperature. When the biscuits are golden brown after 20 min, the trays are removed from the oven and cooled. After cooling, the biscuits were packaged in sachets and then closed with a heat sealer.

### 2.5. Sensory Evaluation of Biscuits

The sensory evaluation was carried out in the University of Maroua. Thirty panelists aged between 18 and 35 years, all of them students of the University of Maroua, participated. They were informed about the objectives and asked not to eat 2 h before. The samples were compared on the basis of the following organoleptic characteristics: taste, color, texture, flavor, and overall acceptability. About 3 g to 5 g of each sample was served to each of the panelists under a white light in a random order. An information sheet was given to them. Between each tasting, they had to rinse their mouths with mineral water and pause for about 2 min in order to avoid appreciation errors. Each attribute was scored based on its intensity scaled on a 9-point hedonic scale (1 = disliked extremely; 2 = disliked very much; 3 = disliked moderately; 4 = disliked slightly; 5 = neither liked nor disliked; 6 = liked slightly; 7 = like moderately; 8 = liked very much; 9 = liked very extremely).

### 2.6. Proximate Composition

The proximate composition of the flours and biscuits was determined according the standard analytical methods (AOAC) [12]. All samples were analyzed in triplicate.

### 2.7. Mineral Determination

The method described by AOAC [13] was used for mineral analysis. The sample was ashed at 550°C and the ash boiled with 10 mL of 20% HCl in a beaker and then filtered into a 100 mL standard flask. The minerals (calcium, magnesium, sodium, potassium, iron copper, zinc, and manganese) were determined by an atomic absorption spectrometer (Varian 220FS SpectrAA, Les Ulis, France). All samples were analyzed in triplicate.

### 2.8. Statistical Analysis

Data were presented as means ± standard deviation (SD). Values were statistically analyzed by one-way analysis of variance (ANOVA test) using IBM SPSS Statistics version 20.0.1 software package, Armonk, New York, USA. Differences were considered significant at *P* < 0.05 using Duncan multiple range test.

## 3. Results and Discussion

### 3.1. Proximate Composition of Different Flours

Table 2 shows the proximate composition of different flours.

The moisture content in the flours ranged from 7.49 (soy bean flour) to 10.58 g/100 g (wheat flour) and was significantly different (*P* < 0.05). The moisture content is a crucial parameter in the storage of flour. Indeed, a moisture content of flour greater than 12% promotes the development of microorganisms. The lower the moisture content of the flour, the more it is possible to hydrate it during kneading to reach an optimal consistency of the dough [14].

The ash content in the flours was in the range of 0.99 (wheat flour) to 2.01/100 g (soy bean flour) and was significantly different (*P* < 0.05). The ash content is an indicator of the purity of the flour. It is related to its extraction rate and the mineralization of the grains milled. It also defines the commercial types of flour, particularly with regard to wheat flour [15].

The fat content in the biscuits ranged from 0.55 for sweet potato flour to 27.3/100 g for soy bean flour and was significantly different (*P* < 0.05). The fat content obtained for sweet potato flour is lower than (1.54%) obtained by Siédogo [16]. This difference may be linked to the variety and production conditions [17].

Protein content in the biscuits varied from 2.57 for sweet potato flour to 36.44 g/100 g for soy bean flour and was significantly different (*P* < 0.05). The protein content of wheat flour (13.48%) is greater than 7%, corresponding to the minimum set by the Codex Alimentarius [18]. The protein content of soy flour (36.51%) used in this study will then make up for the protein deficit in sweet potato and wheat flours.

The fiber content in the biscuits was in the range of 0.56 to 5.01 g/100 g. The lowest fiber content was obtained from wheat flour while the highest was obtained from soy bean flour. The values of fiber content were significantly different (*P* < 0.05). The high fiber content in sweet potato and soy flour may increase the water absorption capacity of the flour.

Carbohydrate content in the biscuits varied from 21.74 for soy bean flour to 83.27 g/100 g for sweet potato flour and was significantly different (*P* < 0.05). Energy content in the biscuits varied from 348.31 for sweet potato to 478.43/100 g for soy bean flour and was significantly different (*P* < 0.05).

### 3.2. Functional Properties

Table 3 shows the functional properties of different flours.

The water absorption capacity of the flours ranged from a low value 73% in control (T_0_) to a high value 84.33% in T4 (100%). Substitution significantly increases the water absorption capacity of the flours (*P* < 0.05). The increase in water absorption capacity would therefore be due to the availability of hydrophilic carbohydrates and functional groups of proteins in flour which are capable of forming hydrogen bonds with water. Indeed, the water absorption capacity depends on the affinity of flour with water. The higher the affinity, the higher the water absorption capacity.

The water solubility index of the flours varied between 17.31 of control (T_0_) and 42.76% of T4 (100%). Substitution also significantly increases the water solubility index of the flours (*P* < 0.05). These results are in agreement with those of Legesse and Admassu [19]. The water solubility index reflects the extent of starch degradation; the increase in solubility index with substitution can be attributed to the difference in granular structure. Indeed, the interior of larger granules should be more difficult to gelatinize due to the limitation of the diffusion of water inside the particle [20].

### 3.3. Different Biscuits Produced

**Figure 1:**
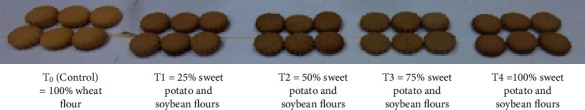
Different biscuits produced.

The different biscuits produced are presented in Figure 1.

The color of the biscuit surface darkens as the substitution rate increases. The color change can be attributed to a higher degree of browning via the Maillard reaction, which is influenced by the water distribution and the reduction of amino acids and sugars [21]. Previous studies have shown that the instrumental measurement of the color of bakery and pastry products is a quality control necessary to determine the influence of an ingredient or the formulation of the product, as well as the storage conditions of the products of bakery [22].

### 3.4. Sensory Evaluation

Table 4 shows the sensory evaluation of biscuits.

Generally, increasing the amount of soybean flour in the biscuits affected their sensory evaluation due to the high levels of protein in soy flour, which affected the taste, color, texture, flavor, and acceptability of the biscuits. These changes in sensory quality were attributed to the formation of hydrogen bonds among the hydroxyl carbonyl, amide hydroxyl groups, and polar groups of the other ingredients of the biscuit flour.

Sensory score of biscuits showed that with regard to taste, color, texture, flavor, and overall acceptability, the sensory characteristics of biscuit T3 (75%) were found to be the best and biscuit T2 (50%) was closer to biscuit T3 (75%). The taste is the primary factor which determines the acceptability of any product, which has the highest impact as far as market success of product is concerned. Biscuit containing 100% soy and sweet potato flours (T4) was rated the poorest in taste (6.26). Biscuit containing 75% soy and sweet potato flours (T3) has the highest mean score (7.61). The mean scores for color of the biscuits changed from 5.4 to 7.19. The highest score (7.19) was also obtained in biscuit T3 (75%). As increase in soy flour, mean score for color was also increasing. This is in contradiction with the result of Banureka and Mahendran [23].

The texture of the crust was related to the external appearance of the biscuit top which implies smoothness or roughness of the crust. The highest score (7.41) was also obtained in biscuit T3 (75%). Except in biscuit T1 (25%) and biscuit T4 (100%), substitution increases the texture of biscuits. In the case of flavor of the biscuits, it was decreased with an increase in the substitution except in biscuit T2 (50%) and T3 (75%). Overall acceptability includes many implications, which is an important parameter in organoleptic estimation. Biscuit T3 (75%) had the highest mean value (7.61), and T1 (25%) had the least mean value (6.23) for the overall acceptability.

### 3.5. Proximate Composition of the Different Most Appreciated Biscuits

Table 5 shows the proximate composition of the most appreciated biscuits.

The moisture content in the biscuits significantly increased with substitution (*P* < 0.05) and varied from 8.62 for T_0_ (control) to 9.59/100 g for T3 (75%). We then note that the incorporation of sweet potato and soy flour in the formulation significantly increases the moisture content. This increase would be the direct consequence of the water absorption capacity of the flours which we have used. Indeed, the functional properties of the compound flours presented above in the characterization of the flours show that the flours composed of wheat-sweet potato-soybeans have a high capacity for absorbing water compared to control and this capacity increases with the substitution.

The ash content in the biscuits also significantly increased with substitution (*P* < 0.05) and varied from 1.36 of biscuit T2 (50%) to 2.02 g/100 g of biscuit T3 (75%). The values obtained are lower compared to the value reported by Eyenga et al. [24] in biscuits (3.55 g/100 g). The fat content in the biscuits significantly increased with substitution (*P* < 0.05) and varied from 2.02 for biscuit T_0_ (control) to 2.55 g/100 g for biscuit T3 (75%). The values obtained were compared to those found by Siedogo [16] in dry biscuits made from wheat (2.143%).

Protein content in the biscuits significantly increased with substitution (*P* < 0.05) and varied from 11.41 for biscuit T_0_ (control) to 13.08 g/100 g for biscuit T3 (75%). This increase could be due to the increase in the proportion of soy flour. The values obtained are higher compared to the value reported by Eyenga et al. [24] in biscuits (10.13 g/100 g). The fiber content in the biscuits significantly increased with substitution (*P* < 0.05) and varied significantly from 1.4 to 4.2 g/100 g. The lowest fiber content was obtained from biscuit T_0_ (control) while the highest was obtained from biscuit T3 (75%). Similar trends in fiber increase in fiber content were also reported by Ayo et al. [25] on the supplementation of malted soy flour on the production of biscuits.

Carbohydrate content in biscuit significantly decreased with substitution (*P* < 0.05). The highest carbohydrate content was observed in biscuit T_0_ (control) (75.02 g/100 g), and the lowest was reported in biscuit T3 (75%) (68.90 g/100 g). A similar trend in decrease in carbohydrate content was also reported by Ayo et al. [26] on the supplementation of malted soy flour on the production of biscuits. The energy content in the biscuits also significantly decreased with substitution (*P* < 0.05) and varied from 349.4 for biscuit T3 (75%) to 363.9 kcal/100 g for biscuit T_0_ (control). Those values are lower compared to 506 kcal/100 g found by Eyenga et al. [24] in biscuits.

### 3.6. Mineral Compositions of the Different Most Appreciated Biscuits

Table 6 shows the mineral compositions of the most appreciated biscuits.

The copper content in the biscuits significantly increased with substitution (*P* < 0.05) and ranged from 0.302 for biscuit T_0_ (control) to 0.374 mg/100 g for biscuit T3 (75%). The copper content increases with substitution. Copper participates in the activity of many enzymes and chemical reactions. It is involved in the oxidation of glucose and is essential for the proper functioning of the myocardium (muscle of the heart). Copper controls bone mineralization and the quality of cartilage and plays a role in the regulation of neurotransmitters, by regulating mood, sleep, memory, and attention. Copper is also involved in the immune process and iron metabolism [26]. The consumption of biscuit T3 (75%) would be a good source of copper with an intake of 0.374 mg/100 g.

The manganese content in the biscuits also significantly increased with substitution (*P* < 0.05) and ranged from 0.908 for biscuit T_0_ (control) to 0.9824 mg/100 g for biscuit T3 (75%). The results obtained are higher compared to those reported by Bello et al. [27]. Manganese plays a role in the formation of hormones including the synthesis of sex hormones and the functioning of the nervous system. Biscuit T3 (75%) is then the most suitable for consumption.

The iron content in biscuits significantly decreases with substitution (*P* < 0.05). Biscuit T_0_ (control) records the highest content (5.89 mg/100 g), and biscuit T3 (75%) records the lowest score (3.456 mg/100 g). The results of this study are comparable to those found by Maria et al. [28] (4.08 mg/100 g) in biscuit made from coconut. Iron also contributes to the formation of red blood cells. It also provides oxygen transport or catalyzes electron transfer reactions, nitrogen fixation for the synthesis of deoxyribonucleic acid [29].

The zinc content also decreases significantly with substitution (*P* < 0.05). Biscuit T_0_ (control) records the highest content (2.127 mg/100 g), and biscuit T3 (75%) records the lowest score (1.509 mg/100 g). The zinc contents are close to those found by Campos-vega [30] (1.4-1.98 mg/100 g). Zinc plays a catalytic and metabolic role by making the active site of nearly 300 enzymes. It also participates in the storage and release of insulin, in the secretion of digestive enzymes [31]. Among the three biscuits, sample T_0_ (control) is the best source of zinc.

The calcium content in the biscuits also significantly increased with substitution (*P* < 0.05) and varied from 30 for biscuit T_0_ (control) from 77 mg/100 g for biscuit T3 (75%). The increase in calcium content with substitution can be explained by the presence of soybeans which 277 mg calcium/100 g [32]. Calcium is involved in the mechanism of muscle contraction, but also in the transmission of nerve impulses. It plays a role in the cascade of blood clotting and in the metabolism of many hormones. A deficiency of calcium provokes in period of growth, the disease corresponds to rickets and in adults to osteomalacia [33]. The biscuits produced from the mixture wheat flour-sweet potato-soy would be beneficial for strengthening bones and reducing calcium deficiencies.

The magnesium content in the biscuits also significantly increased with substitution (*P* < 0.05) and ranged from 32 for biscuit T_0_ (control) to 49 mg/100 g for biscuit T3 (75%). The results obtained are low compared to 62 mg/100 g obtained by Maria et al. [29] in chocolate biscuits. Magnesium is necessary for biochemical reactions in the body, helping to maintain muscle, improving the functioning of the nerve, maintaining the heart rate, and regulating the blood sugar [34]. Biscuit T3 (75%) would then be the most beneficial in terms of magnesium intake.

The potassium content in the biscuits also significantly increased with substitution (*P* < 0.05) and ranged from 184 for biscuit T_0_ (control) to 541 mg/100 g for biscuit T3 (75%). The magnesium content also increases with substitution. The presence of soy and sweet potato in the formulation is responsible for this increase in fact; Badila et al. [17] showed that the sweet potato was rich in potassium with a content of 243 mg/100 MS.

The results of our study are lower compared to those reported by Nisar et al. [35]. Potassium plays an important role in reducing blood pressure. Therefore, biscuit T3 (75%) rich in potassium is recommended to hypertensive subjects.

The sodium content in the biscuits significantly decreased with substitution (*P* < 0.05) and ranged from 94 mg for biscuit T3 (75%) to 254 mg/100 g for biscuit T_0_ (control). A low intake of sodium and sufficient potassium constitutes the dietary challenge that humans have to face daily. High consumption of dietary sodium plays an important role in raising blood pressure [36]. The results of our study are higher compared to those reported by Ponka et al. [37]. Therefore, biscuit T3 (75%) is recommended to hypertensive subjects.

The required daily intake (RDI) values for minerals in foods intended to human consumption were established by the United States Department of Agriculture [38]. The RDI for children is 0.34 mg/day for Cu, 1.2 mg/day for Mn, 7 mg/day for Fe, 3 mg/day for Zn, 500 mg/day for Ca, 80 mg/day for Mg, 3000 mg/day for K, and 1000 mg/day for Na. Consumption of 100 g of biscuit T_0_ (control) would meet 88.83%, 75.67%, 84.15%, 70.9%, 6%, 40%, 6.13%, and 25.4% of the dietary reference intakes, respectively, for copper, manganese, iron, zinc, calcium, magnesium, potassium, and sodium. Consumption of 100 g of biscuit T2 (50%) would meet 96.17%, 79.58%, 51.4%, 56.83%, 7.4%, 50%, 14.46%, and 24.1% of the dietary reference intakes, respectively, for copper, manganese, iron, zinc, calcium, magnesium, potassium, and sodium. Consumption of 100 g of biscuit T3 (75%) would meet 110%, 81.83%, 49.37%, 50.3%, 15.4%, 61.25%, 18.03%, and 9.4% of the dietary reference intakes, respectively, for copper, manganese, iron, zinc, calcium, magnesium, potassium, and sodium. The consumption these biscuits will significantly contribute for improving the RDI of the children.

## 4. Conclusion

Substitution significantly increases the water absorption capacity and the water solubility index of the flours. Biscuit T3 (75%) is the most preferred biscuits. The incorporation of sweet potato and soy flour in the formulation significantly increases the moisture, fat, protein, fiber, copper, manganese, calcium, magnesium, and potassium content in the biscuits. Biscuit T3% (75%) records the highest levels in protein and fiber while biscuit T_0_ (control) records the highest levels of iron, zinc, and sodium. In terms of overall acceptability and nutrient content, biscuit T3 (75%) is the best biscuit. The biscuits produced from the mixture wheat flour-sweet potato-soy would be beneficial for reducing protein and mineral deficiencies.

## Figures and Tables

**Table 1:** Formulation of the biscuits.

Ingredients	T_0_ (control)	T1 (25%)	T2 (50%)	T3 (75%)	T4 (100%)
Wheat flour (g)	500	375	250	125	0
Sweet potato flour (g)	0	100	200	300	400
Soy bean flour (g)	0	25	50	75	100
Margarine (g)	125	125	125	125	125
Sugar (g)	167	167	167	167	167
Eggs (g)	100	100	100	100	100
Yeast (g)	11	11	11	11	11
Vanilla (g)	5	5	5	5	5
Salt (g)	2	2	2	2	2
Water (g)	75	75	75	75	75

T_0_ (control) = 100% wheat flour; T1 = 25% sweet potato and soybean flours; T2 = 50% sweet potato and soybean flours; T3 = 75% sweet potato and soybean flours; T4 = 100% sweet potato and soybean flours.

**Table 2:** Proximate composition of the different flours.

	Soy bean flour	Sweet potato flour	Wheat flour
Moisture (g/100 g)	7.49 ± 0.09^a^	8.43 ± 0.15^a^	10.58 ± 1.01^b^
Ash (g/100 g)	2.01 ± 0.15^c^	1.3 ± 0.09^b^	0.99 ± 0.14^a^
Fat (g/100 g)	27.3 ± 0.18^c^	0.55 ± 0.05^a^	1.44 ± 0.22^b^
Protein (g/100 g)	36.44 ± 0.84^c^	2.57 ± 0.25^a^	13.48 ± 0.1^b^
Fiber (g/100 g)	5.01 ± 0.29^c^	3.87 ± 0.21^b^	0.56 ± 0^a^
Carbohydrate (g/100 g)	21.74 ± 0.97^a^	83.27 ± 0.03^c^	72.94 ± 0.37^b^
Energy (kcal/100 g)	478.43 ± 1.08^c^	348.31 ± 1.33^a^	358.67 ± 3.52^b^

Mean values in the same line with different superscript letters are significantly different (*P* < 0.05).

**Table 3:** Functional property results of different flours (g/100 g).

Flours	Water absorption capacity	Water solubility index
T_0_ (control)	73 ± 11.78^a^	17.31 ± 4.8^a^
T1 (25%)	76.66 ± 10.40^b^	24.69 ± 6.32^b^
T2 (50%)	81 ± 6.55^c^	23.77 ± 7.65^b^
T3 (75%)	81.33 ± 5.033^c^	29.88 ± 1.68^c^
T4 (100%)	84.33 ± 8.5^d^	42.76 ± 11.13^d^

Mean values in the same column with different superscript letters are significantly different (*P* < 0.05). T_0_ (control) = 100% wheat flour; T1 = 25% sweet potato and soybean flours; T2 = 50% sweet potato and soybean flours; T3 = 75% sweet potato and soybean flours; T4 = 100% sweet potato and soybean flours.

**Table 4:** Sensory evaluation of biscuits.

Biscuits	T_0_ (control)	T1 (25%)	T2 (50%)	T3 (75%)	T4 (100%)
Taste	7.3 ± 1.57^bcd^	5.7 ± 1.14^a^	7.6 ± 0.99^d^	7.61 ± 1.0^d^	6.26 ± 1.5^b^
Color	5.4 ± 1.32^a^	5.83 ± 1.1^b^	6.86 ± 1^c^	7.19 ± 0.8^c^	5.8 ± 1.54^ab^
Texture	7.26 ± 0.77^d^	5.46 ± 1.65^a^	7.33 ± 0.57^c^	7.41 ± 0.92^cd^	6.56 ± 1.22^b^
Flavor	7.1 ± 1.37^c^	6.3 ± 1.14^a^	7.23 ± 1.06^bc^	7.58 ± 0.76^c^	6.5 ± 1.79^ab^
Overall acceptability	7.16 ± 1.43^bc^	6.23 ± 1.25^a^	7.2 ± 0.8^bc^	7.61 ± 0.88^c^	6.63 ± 1.60^ab^

Mean values in the same line with different superscript letters are significantly different (*P* < 0.05). T_0_(control) = 100% wheat flour; T1 = 25% sweet potato and soybean flours; T2 = 50% sweet potato and soybean flours; T3 = 75% sweet potato and soybean flours; T4 = 100% sweet potato and soybean flours.

**Table 5:** Proximate composition of the different most appreciated biscuits.

	T_0_ (control)	T2 (50%)	T3 (75%)
Moisture (g/100 g)	8.62 ± 0.02^a^	8.82 ± 0.1^b^	9.59 ± 0.02^c^
Ash (g/100 g)	1.52 ± 0.2^a^	1.36 ± 0.2^a^	2.02 ± 0.02^b^
Fat (g/100 g)	2.02 ± 0.03^a^	2.33 ± 0.01^b^	2.55 ± 0.005^c^
Protein (g/100 g)	11.41 ± 0.14^a^	12.72 ± 0.43^b^	13.08 ± 0.26^b^
Fiber (g/100 g)	1.4 ± 0.07^a^	3.1 ± 0.2^b^	4.2 ± 0.02^c^
Carbohydrate (g/100 g)	75.02 ± 0.03^c^	71.31 ± 0.59^b^	68.90 ± 0.44^a^
Energy (kcal/100 g)	363.9 ± 0.75^c^	358.53 ± 1.23^b^	349.4 ± 0.095^a^

Mean values in the same line with different superscript letters are significantly different (*P* < 0.05). T_0_(control) = 100% wheat flour; T1 = 25% sweet potato and soybean flours; T2 = 50% sweet potato and soybean flours; T3 = 75% sweet potato and soybean flours; T4 = 100% sweet potato and soybean flours.

**Table 6:** Mineral compositions of the different most appreciated biscuits (mg/100 g).

	T_0_ (control)	T2 (50%)	T3 (75%)
Cu	0.302 ± 0.01^a^	0.327 ± 0.01^b^	0.374 ± 0.01^c^
Mn	0.908 ± 0.01^a^	0.955 ± 0.01^b^	0.982 ± 0.01^c^
Fe	5.89 ± 0.01^c^	3.598 ± 0.01^b^	3.456 ± 0.01^a^
Zn	2.127 ± 0.01^c^	1.705 ± 0.01^b^	1.509 ± 0.01^a^
Ca	30 ± 0.5^a^	37 ± 0.5^b^	77 ± 0.5^c^
Mg	32 ± 0.5^a^	40 ± 0.5^b^	49 ± 0.5^c^
K	184 ± 1^a^	434 ± 1^b^	541 ± 1^c^
Na	254 ± 1^c^	241 ± 1^b^	94 ± 1^a^

Mean values in the same line with different superscript letters are significantly different (*P* < 0.05). T_0_(control) = 100% wheat flour; T1 = 25% sweet potato and soybean flours; T2 = 50% sweet potato and soybean flours; T3 = 75% sweet potato and soybean flours; T4 = 100% sweet potato and soybean flours.

## Data Availability

All data are included in the manuscript.
